# Hospital-prepared low-dose atropine eye drops for myopia progression control using atropine sulfate injection diluted in normal saline and lubricants

**DOI:** 10.1186/s13104-022-06240-8

**Published:** 2022-11-05

**Authors:** Nuthida Wongwirawat, Nirachorn Kuchonthara, Sorrawit Boontanomwong, Krit Pongpirul

**Affiliations:** 1 Eye Center, Bumrungrad International Hospital, Bangkok, 10110 Thailand; 2 Pharmaceutical Admixture and Compounding Services, Bumrungrad International Hospital, Bangkok, 10110 Thailand; 3 Clinical Research Center, Bumrungrad International Hospital, Bangkok, 10110 Thailand; 4 Department of Preventive and Social Medicine, Faculty of Medicine, Chulalongkorn University, Bangkok, 10330 Thailand; 5 Department of International Health, Johns Hopkins Bloomberg School of Public Health, Baltimore, MD USA

**Keywords:** Myopia, Eyedrop, Atropine

## Abstract

**Objective:**

As low-dose atropine eye-drops for myopia progression control prepared in-house by diluting the commercial 0.1% atropine eye-drop with sterile water or normal saline has been a common practice whereas atropine injection is readily available and could be a more feasible alternative, this study aimed to assess the properties of the in-house low-dose atropine eye-drops prepared by diluting the atropine injection in two solvents and tested in two temperature conditions.

**Results:**

The 0.01% atropine eye-drops contains no bacteria, fungi, or particulate matter. The levels of atropine sulfate on all samples were comparable to the freshly prepared samples at the 12th week, regardless of the solvents used or storage conditions. The low-dose atropine eye-drops prepared from readily available atropine sulfate injection at healthcare facilities could be an alternative to commercial products.

## Introduction

Myopia or short-sightedness is an ophthalmic condition that leads to blurred vision at distance, generally described by a refractive error of − 0.5 diopter or less and a high myopia of − 5.0 diopter or less [1]. The prevalence of myopia is increasing; as high as 50% of the world population would have some degree of myopia in 2050, of which about 5% will have high myopia which is associated with the risk of irreversible vision impairment and blindness due to pathological changes in the retina, choroid, and sclera [2].

Several behavioral modifications to delay the onset and retard of myopia progression, such as encouraging children to spend time outdoors, and limiting closed-vision activities have been recommended but might not be sufficient nor feasible [3]. Other interventions such as special spectacle or contact lens for myopia progression control also have limited practicality with questionable result in slowing progression of myopia [4].

As a non-selective muscarinic receptor antagonist, atropine has been used as a medical option for slowing myopia progression among children. Despite limited evidence on the exact mechanism of atropine in myopia control, the most plausible mechanism is that it acts directly or indirectly on the retina or scleral, inhibiting thinning or stretching of the scleral, and thereby eye growth [5, 6]. In 2012, Chia et al. demonstrated that atropine 0.01% has comparable myopia control efficacy and minimal side effects compared with atropine at 0.1% and 0.5% [7]. Findings from the Atropine for the Treatment of Myopia studies (ATOM2) and the Low-concentration Atropine for Myopia Progression (LAMP) Study suggested that the low dose atropine eye drop (0.01%, 0.025%, and 0.05%) could significantly slow the myopia progression by 50% with no clinically significant side effects [8, 9]. The low-dose atropine was endorsed by the World Health Organization Western Pacific Region Meeting on Myopia [3].

Several commercial 0.01% atropine eye drops have been approved by the Food and Drug Administration for myopia progression control only in some countries. Hence, in-house preparation by diluting a commercial atropine sulfate eye-drop with sterile water [7, 8, 10, 11] or balanced salt solution (normal saline) has been applied [12, 13]. A practical technique is drawing 0.1 ml of 1% atropine sulfate eye-drop and injecting it into a 10 ml bottle of either commercial artificial tear or balanced salt solution eye-drops. According to the package insert, the original 1% atropine sulfate eye-drop is anticipated to be stable for at least 36 months before opening and only 28 days after opening [12]. The shelf-life is expected to be shorter than one month after opening, which is not practical or economical for clinical practice as well as patient convenience and compliance in the long term.

Alternatively, we anticipated that the atropine sulfate injection commonly used for emergency treatment of bradycardia could also be used for the in-house preparation of the diluted atropine eye-drop for myopia progression control. Similar approach has been accomplished for antifungal (Voriconazole) [11] and immunosuppressive (Cyclosporine A and Tacrolimus) [10] sterile-water-based eye-drops. Atropine sulfate injection is readily available in any healthcare facility whereas the injection formulation usually contains no preservatives which are always of concern in the eye-drop formulation [13]. Moreover, the atropine sulfate injection could be measured and manipulated more easily than atropine sulfate eye-drop. Physical properties of the injection drug could be visually assessed before use whereas the opaque bottle of commercial eye-drop does not allow visual inspection.

As several solvent candidates such as sterile water, normal saline, and lubricant might affect the efficacy of the active substance, introduce some biological contamination, or change the chemical property of the final product, this study aims to investigate the physical, chemical, microbiological properties as well as the shelf-life of the 0.01% atropine eye-drop prepared by using the atropine sulfate injection diluted in two different solvents and tested in two temperature conditions.

## Main text

### Materials and methods

The samples of low-dose atropine sulfate were prepared in normal saline solution (N) and lubricant eye-drops (L). The normal saline-based 0.01% atropine eye-drop was prepared by diluting 5 ml of atropine sulfate injection 0.6 mg/ml (GPO®, Lot J630218, Expiration June 2023) with 25 ml of 0.9% sodium chloride (GHP®, Lot 2010122, Expiration October 2022) to make a total volume of 30 ml then divided into two 15-ml bottles (Table 1). The lubricant-based 0.01% atropine eye-drop was prepared by diluting 2.5 ml of atropine sulfate injection 0.6 mg/ml (GPO®, Lot J630218, Expiration June 2023) with 12.5 ml of oxychloro complex (Purite®)-containing commercial lubricant eye drop (Cellufresh®, Lot T0780, Expiration August 2022) 12.5 ml to make a total volume of 15 ml. All these prepared samples were prepared in an isolator (ESCO®, Model LAC-6A1) at room temperature (15–25 °C) and 40–65% humidity. Then they were allocated at 15 ml into PET bottles sterilized by an ethylene-oxide gas method except samples diluted with Cellufresh® which were prepared bottle by bottle. All the above samples were prepared for 24 bottles of each diluent.

**Table 1:** Low-dose normal saline-based and lubricant-based Atropine Eye-drops

	Normal saline-based	Lubricant-based
Solutes		
• Atropine sulfate injection 0.6 mg/ml	5.0 ml	2.5 ml
Solvents		
• 0.9% sodium chloride 50 ml	25 ml	
• Oxychloro complex (Purite®) 15 ml		12.5 ml
Storage conditions		
• Room temperature (15–25 °C)	NR	LR
• Refrigerator (2–8 °C)	NF	LF

*N normal saline-based, *L* lubricant-based, *R* room temperature, *F* refrigerator

The samples were kept in two storage conditions: room temperature (R) and refrigerator (F) (Sanyo®, Model MPR-720), therefore, four groups underwent the study (NR, NF, LR, LF) (Table 1). All samples were daily dropped for six months to test if air exposure would lead to contamination. The samples underwent biological contamination assessment methods that complied with Chapter 71 of the United States Pharmacopeia (42nd edition). Every 14 days, the remaining fluid of each scheduled opened sample was collected in amber bottles in their corresponding temperature conditions to make 10 mL before sending to the laboratory department, Bumrungrad International Hospital until six months (Table 2). At the 12th week, all samples were sent to perform an assay test at CET-Scientific Services Pte. Ltd., Singapore using liquid chromatography with UV 225 nm detector, 4.6 mm × 15 cm; 5-µm packing L10 column at 40 °C, flow rate 1.2 ml/min, injection volume 20 µl with running time not less than 3 times the retention time of atropine. Pairwise comparison was used to test for statistical differences.

**Table 2:** Levels of the atropine sulfate at the 12th week compared to freshly prepared samples

	NR	NF	LR	LF
#Samples	8	8	8	8
Freshly prepared	0.01224	0.01224	0.01227	0.01227
Week 12	0.01146	0.01086	0.01089	0.01181
Difference	0.00078	0.00138	0.00138	0.00046
p-value	0.231	0.172	0.295	0.295

*N normal saline-based, *L* lubricant-based, *R* room temperature, *F* refrigerator

## Results

The culture of Aerobic bacteria (*Staphylococcus aureus, Bacillus subtilis, Pseudomonas aeruginosa*), anaerobic bacteria (*Clostridium sporogenes*), and fungi (*Candida albicans, Aspergillus brasiliensis, Aspergillus Niger*) had no growth. There was no particulate matter found in all samples and no change in color of all samples. All samples at the 12th week, regardless of the solvents used or storage conditions had comparable levels of the atropine sulfate to the freshly prepared samples (Table 2).

## Discussion

The amount of 0.01% atropine sulfate injection in 0.9% normal saline or lubricant remained in stable quantity to the freshly prepared after three months. No biological contamination or particulate matter was detected within 6 months period in all samples. The findings suggested no significant effect of both solvents or two temperature conditions (room temperature and refrigeration) on remaining amounts of active substance in samples including the effect on physical properties of samples.

Our experiment concurs with previous studies on in-house antifungal [11] and immunosuppressive [10] eye-drop preparations. We also demonstrate similar efficacy of atropine sulfate in various solvents. Sterile water has been a common solvent used in many ophthalmic drug preparations [7, 8, 10, 11] whereas normal saline is increasingly been used [12, 13]. In 2019, Saito et al. demonstrated the physical, chemical, and microbiological stability of the in-house prepared atropine sulfate eye-drops by diluting the 1% atropine sulfate (10 mg/ml) eye-drop with normal saline to have 0.01, 0.1, 0.25, and 0.5% atropine sulfate for as long as 6 months [12].

Our study is the first to comparatively assess the properties of in-house atropine sulfate eye-drops preparation in two different solvents: normal saline and lubricant. The finding is useful supporting evidence for generalizability as the normal saline injection is more commonly available than the sterile water injection and that atropine sulfate injection is easily measured and manipulated as well as readily available in any healthcare facility. The type of solvents seems to have no effects so the clinician could choose the most appropriate solvent for each patient. In some patients who also need lubricating effect, a commercial lubricant eye-drop could be used as a solvent with comparable results.

The in-house preparation of the 0.01% atropine eye-drop would have been assumed to be stable for only 28 days after opening as described in the package insert should the commercial 1% atropine eye-drop be used. The atropine sulfate injection used in our study is proved to be beneficial for practical use as the prepared product could be refilled every three months which is more convenient and economical for the patient than the monthly refill.

## Conclusion

The low-dose atropine eye-drops prepared from readily available atropine sulfate injection at healthcare facilities could be alternative to commercial products.

### Limitation

Several limitations of our study should be noted. Although our study suggested that the shelf life of the product could last for three months after opening in the simulated conditions, this range might be shortened by possible contamination in the real-life use especially in a poor hygiene place. It is advised that the limited contamination of 0.01% atropine eye drops occurred if strict guidelines for dilution using lubricants or normal saline was followed. Longer time should be explored in further study to assess the longest restroration of diluted in-house atropine. Although a recent study suggested that atropine sulfate could be degraded and make the product more acidic and unstable, the findings from the study suggested that the pH of the prepared product of 0.1 mg/ml of atropine remained stable for 6 months [13]. The physical and chemical properties of atropine could have changed after the dilution but there were not formally assessed in our study. Last but not least, the in-house atropine preparation is still not the same as commercial atropine eye drop.

## Data Availability

All data are presented in the manuscript.

## References

[1] Holden BA, Fricke TR, Wilson DA, Jong M, Naidoo KS, Sankaridurg P, Wong TY, Naduvilath TJ, Resnikoff S (2016). Global prevalence of myopia and high myopia and temporal trends from 2000 through 2050. Ophthalmology.

[2] Wong TY, Ferreira A, Hughes R, Carter G, Mitchell P (2014). Epidemiology and disease burden of pathologic myopia and myopic choroidal neovascularization: an evidence-based systematic review. Am J Ophthalmol.

[3] Ang M, Flanagan JL, Wong CW, Muller A, Davis A, Keys D, Resnikoff S, Jong M, Wong TY, Sankaridurg P (2020). Review: myopia control strategies recommendations from the 2018 WHO/IAPB/BHVI Meeting on Myopia. Br J Ophthalmol.

[4] WSPOS Myopia Consensus Statement. https://www.wspos.org/wspos-myopia-consensus-statement/.

[5] Qu J, Zhou X, Xie R, Zhang L, Hu D, Li H, Lu F (2006). The presence of m1 to m5 receptors in human sclera: evidence of the sclera as a potential site of action for muscarinic receptor antagonists. Curr Eye Res.

[6] Tan D, Tay SA, Loh K-L, Chia A (2016). Topical atropine in the control of myopia. Asia Pac J Ophthalmol (Phila).

[7] Chia A, Chua WH, Cheung YB, Wong WL, Lingham A, Fong A, Tan D (2012). Atropine for the treatment of childhood myopia: safety and efficacy of 0.5%, 0.1%, and 0.01% doses (atropine for the treatment of myopia 2). Ophthalmology.

[8] Chia A, Lu QS, Tan D (2016). Five-year clinical trial on atropine for the treatment of myopia 2: myopia control with atropine 0.01% eyedrops. Ophthalmology.

[9] Yam JC, Li FF, Zhang X, Tang SM, Yip BHK, Kam KW, Ko ST, Young AL, Tham CC, Chen LJ, Pang CP (2020). Two-year clinical trial of the low-concentration atropine for myopia progression (LAMP) study: phase 2 report. Ophthalmology.

[10] Ghiglioni DG, Martino PA, Bruschi G, Vitali D, Osnaghi S, Corti MG, Beretta G (2020). Stability and safety traits of novel cyclosporine A and tacrolimus ophthalmic galenic formulations involved in vernal keratoconjunctivitis treatment by a high-resolution mass spectrometry approach. Pharmaceutics.

[11] Roche M, Lannoy D, Bourdon F, Danel C, Labalette P, Berneron C, Simon N, Odou P (2020). Stability of frozen 1% voriconazole eye-drops in both glass and innovative containers. Eur J Pharm Sci.

[12] Saito J, Imaizumi H, Yamatani A (2019). Physical, chemical, and microbiological stability study of diluted atropine eye drops. J Pharm Health Care Sci.

[13] Berton B, Chennell P, Yessaad M, Bouattour Y, Jouannet M, Wasiak M, Sautou V (2020). Stability of ophthalmic atropine solutions for child myopia control. Pharmaceutics.

